# The effects of L-carnitine supplementation on glycemic markers in adults: A systematic review and dose-response meta-analysis

**DOI:** 10.3389/fnut.2022.1082097

**Published:** 2023-01-10

**Authors:** Mohammad Zamani, Naseh Pahlavani, Mahlagha Nikbaf-Shandiz, Niloufar Rasaei, Rasool Ghaffarian-Ensaf, Omid Asbaghi, Farideh Shiraseb, Samira Rastgoo

**Affiliations:** ^1^Department of Clinical Nutrition, School of Nutritional Sciences and Dietetics, Tehran University of Medical Sciences, Tehran, Iran; ^2^Health Sciences Research Center, Torbat Heydariyeh University of Medical Sciences, Torbat-e Heydariyeh, Iran; ^3^Behbahan Faculty of Medical Sciences, Behbahan, Iran; ^4^Student Research Committee, Tabriz University of Medical Sciences, Tabriz, Iran; ^5^Department of Community Nutrition, School of Nutritional Sciences and Dietetics, Tehran University of Medical Sciences (TUMS), Tehran, Iran; ^6^Department of Nutrition, Science and Research Branch, Islamic Azad University, Tehran, Iran; ^7^Cancer Research Center, Shahid Beheshti University of Medical Sciences, Tehran, Iran; ^8^Student Research Committee, Shahid Beheshti University of Medical Sciences, Tehran, Iran; ^9^Department of Cellular and Molecular Nutrition, Faculty of Nutrition Science and Food Technology, National Nutrition and Food Technology Research Institute, Shahid Beheshti University of Medical Sciences, Tehran, Iran

**Keywords:** L-carnitine, glycemic markers, systematic review, meta-analysis, adult, diabetes mellitus type 2

## Abstract

**Background and aims:**

Hyperglycemia and insulin resistance are concerns today worldwide. Recently, L-carnitine supplementation has been suggested as an effective adjunctive therapy in glycemic control. Therefore, it seems important to investigate its effect on glycemic markers.

**Methods:**

PubMed, Scopus, Web of Science, and the Cochrane databases were searched in October 2022 for prospective studies on the effects of L-carnitine supplementation on glycemic markers. Inclusion criteria included adult participants and taking oral L-carnitine supplements for at least seven days. The pooled weighted mean difference (WMD) was calculated using a random-effects model.

**Results:**

We included the 41 randomized controlled trials (RCTs) (*n* = 2900) with 44 effect sizes in this study. In the pooled analysis; L-carnitine supplementation had a significant effect on fasting blood glucose (FBG) (mg/dl) [WMD = −3.22 mg/dl; 95% CI, −5.21 to −1.23; *p* = 0.002; *I*^2^ = 88.6%, *p* < 0.001], hemoglobin A1c (HbA1c) (%) [WMD = −0.27%; 95% CI, −0.47 to −0.07; *p* = 0.007; *I*^2^ = 90.1%, *p* < 0.001] and homeostasis model assessment-estimate insulin resistance (HOMA-IR) [WMD = −0.73; 95% CI, −1.21 to −0.25; *p* = 0.003; *I*^2^ = 98.2%, *p* < 0.001] in the intervention compared to the control group. L-carnitine supplementation had a reducing effect on baseline FBG ≥100 mg/dl, trial duration ≥12 weeks, intervention dose ≥2 g/day, participants with overweight and obesity (baseline BMI 25–29.9 and >30 kg/m^2^), and diabetic patients. Also, L-carnitine significantly affected insulin (pmol/l), HOMA-IR (%), and HbA1c (%) in trial duration ≥12 weeks, intervention dose ≥2 g/day, and participants with obesity (baseline BMI >30 kg/m^2^). It also had a reducing effect on HOMA-IR in diabetic patients, non-diabetic patients, and just diabetic patients for insulin, and HbA1c. There was a significant nonlinear relationship between the duration of intervention and changes in FBG, HbA1c, and HOMA-IR. In addition, there was a significant nonlinear relationship between dose (≥2 g/day) and changes in insulin, as well as a significant linear relationship between the duration (weeks) (coefficients = −16.45, *p* = 0.004) of intervention and changes in HbA1C.

**Conclusions:**

L-carnitine could reduce the levels of FBG, HbA1c, and HOMA-IR.

**Systematic review registration:**

https://www.crd.york.ac.uk/prospero/, identifier: CRD42022358692.

## Introduction

Hyperglycemia has increased dramatically in the last two decades. A combination of obesity, reduced activity levels, and aging may have contributed to this disorder (1). Another glycemic disorder is insulin resistance (IR) which muscles, fat, and liver cells do not respond to insulin effectively (2). Among adults worldwide, IR occurs in 15.5–46.5% of cases (3). Glycemic markers including fasting blood glucose (FBG), hemoglobin A1c (HbA1c), insulin, and Homeostatic Model Assessment for Insulin Resistance (HOMA-IR) are used to monitor glycemic control in clinical practice (4). Glycemic markers are associated with chronic and metabolic diseases such as cardiovascular diseases (5–7). In different studies, the rate of poor control of glycemic markers has been estimated as 41.6% (8), 54.8% (9), and 86.2% (10). Various factors are effective in controlling and improving glycemic markers, such as improving lifestyle, physical activity, changing dietary intake, and of course some nutritional supplement consumption (11–14).

L-carnitine is involved in transports long-chain fatty acids from the cytoplasm to the mitochondria, as well as the beta-oxidation of fatty acid indirectly; therefore, it contributes to fat metabolism (15). Moreover; L-carnitine maintains insulin sensitivity and metabolic flexibility (16).

Any L-carnitine deficiency may lead to disturbances in fat and glucose metabolism. L-carnitine affects glycemic markers, hyperglycemia, and insulin resistance (17–20). In summary, L-carnitine may reduce insulin resistance by removing harmful lipids from cells, mending cell membranes, and reducing accumulated acyl CoA derivatives and/or their metabolites (18, 20). L-carnitine influences glycemic control *via* a number of different pathways, including the following:

(1) Controlling the pyruvate dehydrogenase complex's (PDHC) activity and the intramitochondrial acetyl-CoA/CoA ratio; (2) Modifying the expression of glycolytic and gluconeogenic enzymes; (3) altering the gene expression in the insulin signaling cascade; and (4) activating the IGF-1 axis and cascade of IGF-1 signaling (21).

Several studies have examined the impact of L-carnitine on glycemic markers. For instance, according to a study conducted in 2022 by Nejati et al., taking L-carnitine orally led to a significant decrease in insulin, FBG, HOMA-IR, and insulin sensitivity (22). But, Liang et al. demonstrated that taking L-carnitine daily for 12 weeks did not affect FBG, HbA1c, and insulin (23). A meta-analysis of 37 RCTs conducted by Fathizadeh et al., in 2019 has shown that taking L-carnitine results in a decrease in FBG, HOMA-IR, HbA1c, and insulin (24). Moreover, the meta-analysis of 24 RCTs conducted by Asadi et al., in 2020 has shown that L-carnitine supplementation could reduce glycemic markers like FBG, HbA1c, and HOMA-IR. However, insulin was not investigated in this study (25).

Consequently, studies on the effect of L-carnitine supplementation and glycemic markers have shown different controversial findings based on studies and due to several factors, including dosage, duration, types of carnitines, and use of L-carnitine with other supplements intervention. Although, a meta-analysis appeared in 2019 (24). However, several RCTs have been published since then, so we aimed to update and perform further analysis such as linear and non-linear dose-response and further subgroup analysis to find more data for interpretation. Therefore; we conducted a meta-analysis on the effect of L-carnitine on glycemic markers considering with comprehensive point of view on glycemic markers in adults.

## Materials and methods

In the current study, the preferred reporting items for systematic reviews and meta-analyses (PRISMA) declaration was used (26). This study was registered in PROSPERO (CRD42022358692).

### Search strategy

As part of our systematic literature search, we searched PubMed, Scopus, Web of Science, and the Cochrane databases for randomized control trials (RCTs) on the effects of L-carnitine supplementation on glycemic markers published up to October 2022. A bibliography of relevant studies, including prior meta-analyses (27), was reviewed to identify potential missing studies. Neither the length nor language of publications were restricted. To search for all items related to L-carnitine supplementation and glycemic markers, we used a search framework, namely, PICO (Participant, Intervention, Comparison/Control, and Outcome) as explained in the study selection. We used Mesh and non-Mesh terms to search the literature as follows: “Vitamin BT” OR “L-carnitine” OR “carnitine” OR “levocarnitine” OR “bicarnesine” OR “L-acetylcarnitine” OR “acetyl-L-carnitine” AND “Intervention” OR “Intervention Study” OR “Intervention Studies” OR “controlled trial” OR randomized OR random OR randomly OR placebo OR “clinical trial” OR Trial OR “randomized controlled trial” OR “randomized clinical trial” OR RCT OR blinded OR “double blind” OR “double blinded” OR trial OR “clinical trial” OR trials OR “Pragmatic Clinical Trial” OR “Cross-Over Studies” OR “Cross-Over” OR “Cross-Over Study” OR parallel OR “parallel study” OR “parallel trial.”

### Study selection

We included studies that meet the inclusion criteria as follows: (1) RCTs (parallel or cross-over); (2) used oral intake of L-carnitine; (3) evaluate the effects of L-carnitine supplementation on FBG, HbA1c, insulin, and HOMA-IR; (4) intervention duration was at least seven days (we considered RCTs as separate studies if they were with two or more eligible arms); (5) used adult participants (≥18 years old); (6) used means and standard deviations (SDs) for FBG (mg/dl), HbA1c (%), insulin (pmol/l), and HOMA-IR, or any other effect sizes that were possible for calculating the mean and SD. We searched human studies without any language restrictions. Screening of the title and abstracts of the included studies by extracting the results and assessing the validity of the studies were performed independently by two authors (SR and OA) to determine whether they were eligible or not. Disputes and differences were settled with discussion. Studies were excluded if they met the following exclusion criteria: studies conducted on children, adolescents, or animals, reviews studies, *in vitro* studies, editorial papers, gray literature, books, conference abstracts, and RCTs conducted without a placebo or control group. Additionally, studies in which L-carnitine was administered by infusion, consumed for <7 days, or combined with vitamins or minerals were excluded.

### Data extraction

Separate re-checks were conducted on all eligible RCTs, and two independent investigators (MZ and MN) extracted the following information. Several factors were extracted for further analysis, including the name of the first author, country, publication year, type of clinical trial, participants' characteristics (mean age, BMI, sex), randomization, blinding, sample size, number of interventions, and control group participants, form and dose of L-carnitine supplementation, study duration, and related details. The mg/day L-carnitine dosages were converted to g/day. For both parallel and cross-over trials, we collected the mean and SD for FBG (mg/dl), HbA1c (%), insulin (pmol/l), and HOMA-IR. In the absence of this data, we subtracted the mean value at baseline from the mean value at the end of the study for calculating the mean difference.

### Quality assessment

An assessment of the quality of the studies was conducted using the Cochrane Collaboration tool (28). We assessed all studies for several sources of biases such as randomized sequence generation, concealment of allocation, blinding of participants and staff, inadequate outcome data, selective reporting, and others. Finally, three groups of risk of bias were created: high risk of bias, moderate risk of bias, and low risk of bias. We had two reviewers (FS and NP) evaluate the quality of the work independently, and we settled any conflicting opinions through discussion.

### Statistical analysis

Stata 11.0 was used to conduct the statistical analysis (Stata Corp, College Station, TX). *P*-values of 0.05 were deemed statistically significant for all two-tailed tests. The pooled weighted mean difference (WMD) was computed using a random-effects model to take into account any existing heterogeneity (29). We assessed the mean differences in FBG, HbA1c, insulin, and HOMA-IR between the L-carnitine supplementation and control groups from the baseline to the post-intervention. The following equation was used to determine the SD of the mean difference: SD = square root [(SD at baseline)^2^+(SD at the end of study)^2^ −(2 r × SD at baseline × SD at the end of study)] (30). In each study that reported standard errors (SE) rather than SD, we converted the SEs, 95 percent confidence intervals (CIs), and interquartile ranges (IQRs) to SDs using the Hozo et al. approach. The SD was calculated using the formula SD = SE × √n (where n is the total number of participants in each group) (31). The correlation coefficient was set at 0.8 for r (32). To identify the cause of heterogeneity, a subgroup analysis was carried out. According to the criteria outlined by Fu et al., where there should be at least 6–10 studies for continuous and a minimum of 4 studies for categorical subgroup variables, subgroups were chosen based on the necessary minimum number of studies (33, 34). Other subgroup analyses were conducted based on baseline BMI [overweight (25–29.9 kg/m^2^) and obese (>30 kg/m^2^)] as well as trial duration (<12 and ≥12 weeks), intervention dose (<2 and ≥2 g/day), and health status (diabetic, non-diabetic). The statistical heterogeneity was assessed in the meta-analyses using the *I*^2^ or Cochrane's *Q* test (35), with values higher than 40% indicating strong heterogeneity (36).

The funnel plot test and Egger's test and Begg's test were used to examining publication bias (37, 38). The leave-one-out method (i.e., removing one trail at a time and recalculating the impact size) was used to examine the impact of each study on the pooled effect size. Sensitivity analysis was carried out to determine how many inferences were dependent on a particular sample. In order to identify and mitigate the effects of publication bias, we used the trim-and-fill method (39). The potential impact of L-carnitine (g/d) dosage and duration on FBG, HbA1c, insulin, and HOMA-IR was evaluated using meta-regression. Additionally, we used a non-linear regression model to deal with the synthesis of the correlated dose-response data from various studies for the dose-response analysis between L-carnitine supplementation and FBG, HbA1c, insulin, and HOMA-IR. This model focuses on inverse variance weighted least squares regression and cluster robust error variances (40, 41).

### Certainty assessment

Using the GRADE (Grading of Recommendations Assessment, Development, and Evaluation) method, which was previously discussed, the overall degree of evidence certainty across the studies was evaluated and summarized (42).

## Results

### Study selection

**Figure 1:**
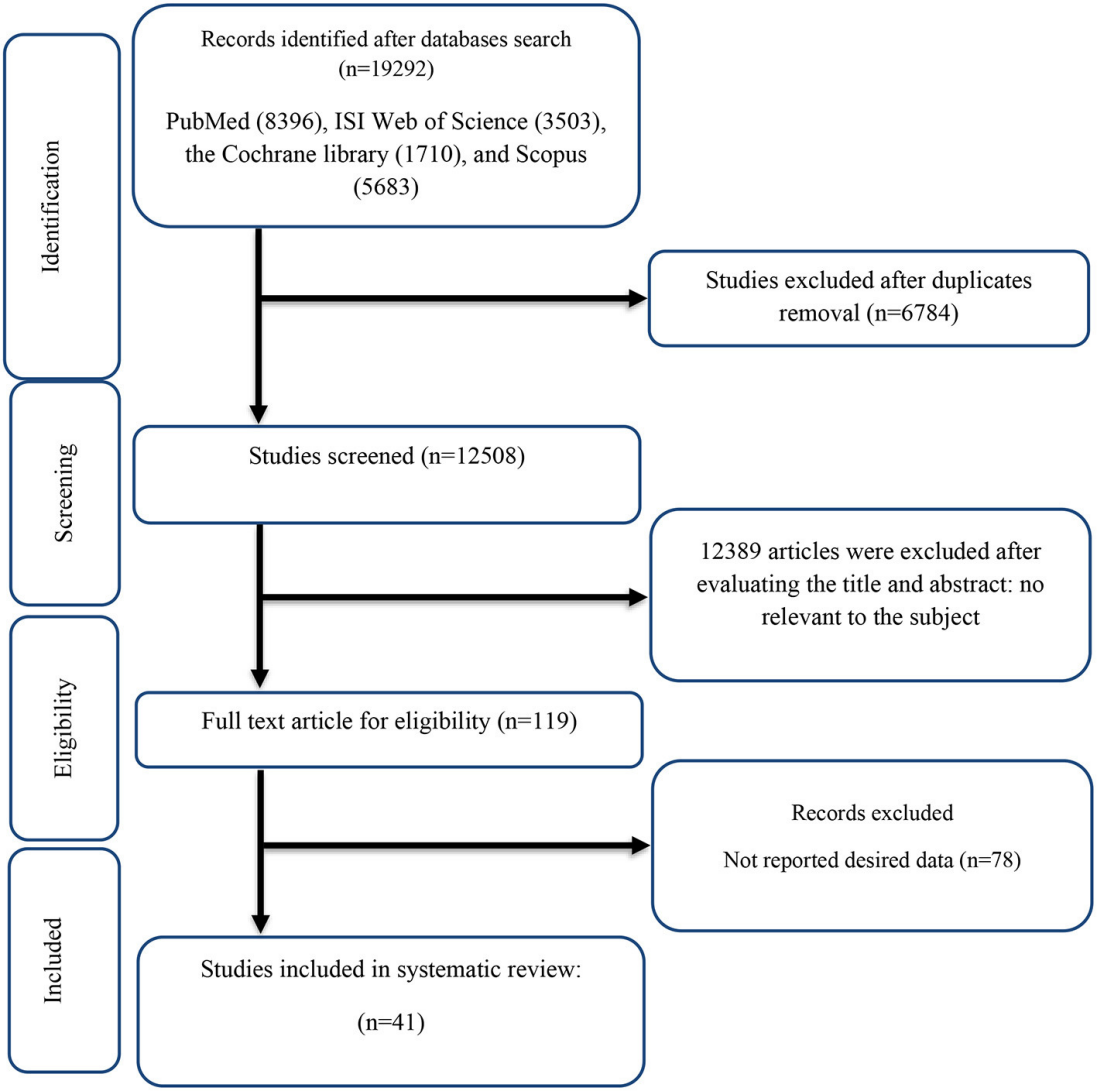
Flow chart of study selection for inclusion trials in the systematic review.

The flow chart of the study was presented in Figure 1 and we described the selection process and the references retrieved from the database in this figure. We identified in the first step of the electronic databases search a total number of 19,292 studies. We excluded duplicated (*n* = 6,784) and irrelevant studies (*n* = 12,389) based on titles and abstracts, and 119 full-text relevant articles were reviewed. A total of 78 studies were excluded due to the following reasons: insufficient outcome data reported, acute oral ingestion, or short duration of supplementation (<1 week). Finally, we included a total of 41 studies (16, 17, 23, 43–80) with 44 effect sizes included in the qualitative synthesis.

### Study characteristics

The study design characteristics showed in Table 1. A summary of the risk of bias assessment of the included studies is presented in Table 2.

**Table 1:** Characteristic of included studies in the meta-analysis.

**References**	**Country**	**Study design**	**Participant**	**Sample size and sex**	**Sample size**		**Trial duration (week)**	**Means age**		**Means BMI**		**Intervention**		**Adverse events**
					**IG**	**CG**		**IG**	**CG**	**IG**	**CG**	**Carnitine (g/d)**	**Control group**	
Liang et al. (23)	China	Parallel, R, PC, DB	Diabetic patient	M/F (F: 30, M: 16)	23	23	12	59.4 ± 1.7	57.9 ± 2.6	27.2 ± 3.1	26.9 ± 2.8	3	Placebo	NR
Derosa et al. (53)	Italy	Parallel, R, PC, DB	Type 2 diabetes mellitus	M/F (F: 47, M: 47)	46	48	24	52 ± 6	50 ± 7	27.3 ± 2.5	26.8 ± 2.2	2	Placebo	No adverse events
Rahbar et al. (17)	Iran	Parallel, R, PC, DB	Type 2 diabetes mellitus	M/F (F: 13, M: 22)	19	16	12	50.5 ± 4.8	52.2 ± 2.6	27.9 ± 2	28.2 ± 1.52	3	Placebo	No adverse events
Santo et al. (77)	Italy	Parallel, R, PC, DB	Type 2 diabetes	M/F: 74	37	37	12	61.75 ± 3.03	61.26 ± 1.6	34 ± 0.02	36 ± 0.01	2	Placebo	NR
Malaguarnera et al. (65)	Italy	Parallel, R, PC, DB	Centenarians	M/F (F: 45, M: 21)	32	34	24	101 ± 1.3	101 ± 1.4	22.2 ± 4.7	22.6 ± 4.1	2	Placebo	NR
González-Ortiz et al. (45)	Mexico	Parallel, R, PC, DB	Type 2 diabetes mellitus patients	M/F (F: 6, M: 6)	6	6	4	44.1 ± 7.3	42.6 ± 9.2	27.2 ± 2.7	27.8 ± 2.7	3	Placebo	No adverse events
Delaš et al. (52)	Croatia	Parallel, R, PC, DB	Sedentary population	M/F (F: 18, M: 12)	18	12	2	23.1 ± 3.5	21.3 ± 2.6	22.7 ± 2.7	23.8 ± 5.2	2	Placebo	NR
Bloomer et al. (50)	USA	Parallel, R, PC, DB	Pre-diabetics	M/F: 29	14	15	8	31 ± 12	35 ± 12	28.5 ± 7.6	31.7 ± 8.4	3	Placebo	No adverse events
Malaguarnera et al. (67)	Italy	Parallel, R, PC	Patients with diabetes	M/F (F: 23, M: 58)	41	40	12	49 ± 13	48 ± 11	27.5 ± 1.8	27.4 ± 1.8	2	Placebo	Gastrointe stinal tract complaints
Galvano et al. (58)	Italy	Parallel, R, PC, DB	Type 2 diabetes mellitus	M/F (F: 45, M: 30)	38	37	16	52.1 ± 8.1	51.4 ± 7.6	27.8 ± 2	27.1 ± 2.4	2	Control diet	No adverse events
Malaguarnera et al. (66)	Italy	Parallel, R, PC	Type 2 diabetes mellitus	M/F (F: 25, M: 55)	40	40	12	47 ± 13	45 ± 12	26.8 ± 1.3	26.5 ± 1.7	2	Control diet	NR
Bloomer et al. (74)	USA	Parallel, R, PC, DB	Untrained, normolipidemic subjects	Arm a: M/F: 20	Arm a: 10	10	8	Arm a: 26.1 ± 6.65	28.25 ± 6.01	Arm a: 26.72 ± 3.9	27.24 ± 6.18	Arm a: 1		No adverse events
				Arm b: M/F: 22	Arm b: 12			Arm a: 26.56 ± 4.4		Arm a: 24.47 ± 5.4		Arm a: 3		
Mojtaba et al. (69)	Iran	Crossover, R, PC, DB	Healthy males	M: 30	30	30	3	18–24	18–24	NR	NR	3	Placebo	NR
Malaguarnera et al. (68)	Italy	Parallel, R, PC, DB	Nonalcoholic steatohepatitis	M/F (F: 34, M: 40)	36	38	24	47.9 ± 5.4	47.8 ± 5.8	26.6 ± 3.7	26.5 ± 3.8	2	Control diet	NR
Molfino et al. (70)	Italy	Parallel, R, PC	Patients with impaired glucose metabolism	M/F (F: 4, M: 12)	8	8	2	69.1 ± 12.6	64.2 ± 14.5	28.6 ± 6.8	25.8 ± 6.8	4	Placebo	No adverse events
Derosa et al. (55)	Italy	Parallel, R, PC, DB	Diabetic patients	M/F (F: 126, M: 128)	129	125	52	54 ± 5	51 ± 4	33.9 ± 3.5	33.4 ± 3.2	2	Control diet	No adverse events
Derosa et al. (54)	Italy	Parallel, R, PC, DB	Obese diabetic patients	M/F (F: 131, M: 127)	132	126	52	51 ± 4	53 ± 6	32.9 ± 2.8	33.1 ± 2.9	2	Control diet	Flatulence, Constipation, Abdominal pain, Fatty/oily evacuation, Increased defecation, Fecal urgency, Malaise
Wall et al. (76)	United Kingdom	Parallel, R, PC, DB	Athletes	M/F: 14	7	7	24	27.1 ± 6	24.6 ± 6	23.9 ± 2.7	22.2 ± 2.1	2	Control diet	NR
Rafraf et al. (73)	Iran	Parallel, R, PC, DB	Obese women	F: 22	Arm a: 11	11	8	Arm a: 34.4 ± 5.48	Arm a: 36.5 ± 7.3	Arm a: 33.99 ± 2.33	33.52 ± 2.04	2	Placebo	NR
					Arm b: 11			Arm b: 34.8 ± 6.25	Arm b: 36.5 ± 7.3	Arm b: 33.33 ± 2.46		2 + EXR	Placebo and EXR	
Hlais et al. (60)	Lebanon	Parallel, R, PC	Hypertrigl yceridemia	M/F: 34	15	19	12	55.6 ± 10.7	51.79 ± 12.31	31.34 ± 5.42	29.93 ± 3.8	1	Placebo	NR
Odo et al. (80)	Japan	Parallel, R, PC, DB	Overweight males	Arm a: M: 10	Arm a: 5	5	4	Arm a: 44.4 ± 3.5	Arm a: 40.2 ± 4.8	Arm a: 26.6 ± 1.1	Arm a: 26.2 ± 1	Arm a: 0.5	Placebo combination with motivation training	NR
				Arm a: M: 11	Arm a: 6			Arm b: 43.3 ± 8.2	Arm b: 43.4 ± 6.5	Arm a: 25.8 ± 0.6	Arm a: 26.4 ± 0.6	Arm a: 0.5	Placebo	
Bonomini et al. (51)	Italy	Parallel, R, PC, SB	Patients treated with CAPD	M/F (F: 16, M: 19)	21	14	16	56 ± 13	62 ± 12	26 ± 4	28 ± 5	2	Control diet	No adverse events
Hong et al. (61)	Korea	Parallel, R, PC, DB	NAFLD and impaired glucose metabolism	M/F (F: 16, M: 36)	26	26	12	51.5 ± 9.4	52 ± 9.6	27.2 ± 2.6	27 ± 3.1	1	Control diet	Musculoske letal pain and gastrointes tinal disturbance
Dehghan Banadaki et al. (62)	Iran	Parallel, R, PC	Hemodialysis patients	M/F (F: 20, M: 30)	25	25	12	63.4 ± 12.9	62.1 ± 10.2	24.4 ± 3.4	24.6 ± 3	1	Control diet	NR
Bae et al. (49)	Korea	Parallel, R, PC, DB	Patients with diabetes and NAFLD	M/F (F: 24, M: 54)	39	39	12	50.6 ± 9.3	52 ± 9.4	28.2 ± 2.6	26.7 ± 3.7	2.5	Placebo	No adverse events
Mosah et al. (46)	Iraq	Parallel, R, PC, SB	Obese females	F: 36	18	18	12	33.11 ± 6.53	32.72 ± 7	34.58 ± 2.77	34.83 ± 2.99	1	Control diet	NR
Ramazanpour et al. (78)	Iran	Parallel, R, PC	Diabetic patients	M: 20	10	10	4	51.6 ± 2.98	50.8 ± 2.2	24.7 ± 0.6	25.5 ± 0.59	0.5	Control diet	NR
Samimi et al. (72)	Iran	Parallel, R, PC, DB	Polycystic ovary syndrome	F: 60	30	30	12	24.8 ± 5.5	25.5 ± 5.7	29.1 ± 3.4	28.9 ± 3.9	0.25	Placebo	NR
An et al. (48)	Korea	Parallel, R, PC, DB	Patients with hypothyroidism on levothyroxine treatment	M/F (F: 50, M: 3)	28	25	12	49 ± 8.2	50.9 ± 9.1	24.7 ± 3.1	22.7 ± 2.8	1.8	Placebo	Nausea, generalized edema, epigastric discomfort
Alavinejad et al. (79)	Iran	Parallel, R, PC, DB	Non-alcoholic fatty liver disease	M/F (F: 16, M: 38)	28	26	12	60 ± 5	59 ± 9	28.6 ± 4.6	29.5 ± 3.6	2.25	Control diet	NR
Ghorbani et al. (63)	Iran	Parallel, R, PC, DB	Type 2 diabetic women	F: 20	10	10	8	52.7 ± 1.6	52.7 ± 1.6	29.82 ± 4.35	29.82 ± 4.35	0.5	Placebo	NR
Parvanova et al. (71)	Italy	Parallel, R, PC, DB	Type 2 diabetes	M/F (F: 64, M: 165)	116	113	24	64.9 ± 7.7	64.6 ± 7.5	30 ± 4.7	30 ± 5	2	Placebo	No adverse events
Hassani et al. (59)	Iran	Parallel, R, PC, DB	Type 2 diabetic women	F: 20	10	10	8	52.2 ± 6.8	53.6 ± 3.2	29.36 ± 4.34	28.41 ± 4.06	0.5	Placebo	NR
Mahdavi et al. (64)	Iran	Parallel, R, PC, DB	Knee osteoarthritis	F: 48	23	25	8	51.56 ± 6.24	52.6 ± 7.1	33.12 ± 2.15	33.64 ± 2.41	0.75	Placebo	NR
El-Sheikh et al. (57)	Egypt	Parallel, R, PC	Type 2 diabetic patients	M/F (F: 39, M: 19)	31	27	24	50.9 ± 8.6	50.3 ± 8.8	34.46 ± 5.3	34.25 ± 5.6	2	Control diet	No adverse events
El Sharkwy et al. (56)	Egypt	Parallel, R, PC, DB	Polycystic ovary syndrome	F: 162	80	82	12	26.2 ± 2.8	26.6 ± 1.5	29.7 ± 2.4	29.5 ± 3.3	1.8	Control diet	NR
Sharkwy et al. (44)	Egypt	Parallel, R, PC, DB	Obese polycystic ovary syndrome	F: 274	138	136	13	25.7 ± 1.7	26.1 ± 2.2	35.5 ± 3.2	34.4 ± 3.4	3	Placebo	NR
Bruls et al. (16)	Netherlands	Crossover, R, PC, DB	Volunteers with impaired glucose tolerance	M/F (F: 3, M: 20)	11	12	4	62 ± 6.8	61 ± 6.9	29.7 ± 1.6	28.9 ± 2.1	2	Placebo	NR
Sepandar et al. (43)	Iran	Parallel, R, PC, DB	Pemphigus vulgaris	M/F (F: 32, M: 20)	26	26	8	41.04 ± 9.65	40.65 ± 9.9	28.07 ± 4.33	26.66 ± 3.48	2	Placebo	No adverse events
Tauqir et al. (75)	Pakistan	Parallel, R, PC, DB	Polycystic ovary syndrome	F: 147	72	75	12	26.96 ± 6.31	25.23 ± 6.06	31.08 ± 4.77	28.27 ± 5.15	1.5	Control diet	NR
AbuMoh'd et al. (47)	Jordan	Parallel, R, PC, DB	Trained-endurance athletes	M: 20	10	10	3	22.13 ± 2.66	21.63 ± 2.23	21.89 ± 0.57	22.19 ± 0.47	3	Placebo	NR

IG, intervention group; CG, control group; DB, double-blinded; SB, single-blinded; PC, placebo-controlled; CO, controlled; RA, randomized; NR, not reported; F, Female; M, Male; NR, not reported.

**Table 2:** Risk of bias assessment.

**References**	**Random sequence generation**	**Allocation concealment**	**Selective reporting**	**Other sources of bias**	**Blinding (participants and personnel)**	**Blinding (outcome assessment)**	**Incomplete outcome data**	**General risk of bias**
Liang et al. (23)	U	H	H	H	L	U	L	Bad
Derosa et al. (53)	L	H	H	H	L	U	L	Bad
Rahbar et al. (17)	L	H	H	H	L	U	L	Bad
Santo et al. (77)	L	H	H	H	L	U	L	Bad
Malaguarnera et al. (65)	L	L	H	H	L	U	L	Fair
González-Ortiz et al. (45)	L	U	H	H	H	L	L	Bad
Delaš et al. (52)	L	H	H	H	L	U	L	Bad
Bloomer et al. (50)	L	H	L	H	L	U	L	Fair
Malaguarnera et al. (67)	L	H	H	H	H	H	L	Bad
Galvano et al. (58)	L	H	H	L	L	U	L	Fair
Malaguarnera et al. (66)	L	H	H	H	H	H	L	Bad
Bloomer et al. (74)	L	H	H	L	L	U	L	Fair
Mojtaba et al. (69)	L	H	H	H	L	U	L	Bad
Malaguarnera et al. (68)	L	L	H	H	L	U	L	Fair
Molfino et al. (70)	L	H	H	H	H	H	L	Bad
Derosa et al. (55)	L	H	L	H	L	U	L	Fair
Derosa et al. (54)	L	H	L	L	L	U	L	Good
Wall et al. (76)	L	H	H	H	L	U	L	Bad
Rafraf et al. (73)	L	H	H	H	L	U	L	Bad
Hlais et al. (60)	L	H	H	H	H	H	H	Bad
Odo et al. (80)	U	H	H	H	L	U	L	Bad
Bonomini et al. (51)	L	L	H	H	H	H	L	Bad
Hong et al. (61)	L	L	L	L	L	U	L	Good
Dehghan Banadaki et al. (62)	L	H	H	H	H	H	L	Bad
Bae et al. (49)	L	H	H	H	L	U	H	Bad
Mosah et al. (46)	L	H	H	H	H	H	L	Bad
Ramazanpour et al. (78)	L	H	H	H	H	H	L	Bad
Samimi et al. (72)	L	L	H	L	L	U	L	Good
An et al. (48)	L	L	H	H	L	U	L	Fair
Alavinejad et al. (79)	L	L	H	H	L	U	L	Fair
Ghorbani et al. (63)	L	H	L	H	L	U	L	Fair
Parvanova et al. (71)	L	L	L	L	L	U	L	Good
Hassani et al. (59)	L	H	H	H	L	U	L	Bad
Mahdavi et al. (64)	L	L	H	L	L	U	L	Good
El-Sheikh et al. (57)	L	L	L	L	L	U	L	Good
El Sharkwy et al. (56)	L	L	H	L	L	U	L	Good
Sharkwy et al. (44)	L	L	H	H	L	U	L	Fair
Bruls et al. (16)	L	H	H	H	L	U	L	Bad
Sepandar et al. (43)	L	L	H	L	L	U	L	Good
Tauqir et al. (75)	L	L	H	H	L	U	L	Fair
AbuMoh'd et al. (47)	L	H	H	H	L	U	L	Bad

U; unclear risk of bias, L; low risk of bias, H; high risk of bias.

Good < 2 high risk of bias; Fair = 2 high risk of bias; Bad > 2 high risk of bias.

The supplementation duration of included studies ranged from 2 to 52 weeks. The daily dosage of L-carnitine supplementation ranged from 0.25 to 4 g/day. 39 parallel (17, 23, 43–68, 70–80) and 2 cross-over (16, 69) studies were included in this study. The mean age ranged from 18–101 years and baseline BMI of included studies ranged from 22.19 to 36 kg/m^2^ in the intervention group, respectively. Thirteen studies included only males or females (46, 56, 59, 63, 64, 69, 72, 73, 78, 80), and 28 included both sexes (16, 17, 23, 43, 45, 48–55, 57, 58, 60–62, 65–68, 70, 71, 74, 76, 77, 79).

The investigation by Liang et al. (23) had two types of participants (outpatients and inpatients with non-insulin-dependent diabetes mellitus) so we considered two arms for this study. The investigation by Bloomer et al. (74) had two types of intervention doses (1 and 3 g/day) so we considered two arms for this study. Also, Rafraf et al. (73) had two types of intervention (L-carnitine supplementation + aerobic training and L-carnitine supplementation) so we considered two arms for this study. In addition, Odo et al. (80) had two types of control groups (Placebo Combination with Motivation Training and Placebo) so we considered two arms for this study.

Out of 41 RCTs, 39 studies have shown a significant lowering effect of L-carnitine supplementation on FBG (mg/dl) (16, 17, 23, 43, 45–69, 71–80), 19 studies on serum insulin (pmol/l) (16, 43, 44, 51, 53–55, 57, 59, 61, 63, 68, 71–76, 80), 20 studies on serum HbA1c (%) (16, 17, 23, 45, 48, 49, 53–55, 57, 58, 61, 63, 66, 67, 71, 74, 77, 79, 80), and 17 studies on HOMA-IR (43, 44, 49, 50, 54–57, 59, 61, 63, 68, 70–73, 75).

### Adverse events

Adverse effects were mentioned in the studies by Derosa et al. (Flatulence, Constipation, abdominal pain, fatty/oily evacuation, increased defecation, fecal urgency, malaise), Hong et al. (61) (musculoskeletal pain and gastrointestinal disturbance), Malaguarnera et al. (67) (gastrointestinal tract complaints) and An et al. (48) (nausea, generalized edema, epigastric discomfort).

### Qualitative data assessment

The qualitative data based on the Cochrane risk of bias assessment tool were presented in Table 2. Eight studies had low risk of bias (43, 55–57, 61, 64, 71, 72), 11 studies had moderate risk of bias (44, 48, 50, 54, 58, 63, 65, 68, 74, 75, 79) and 22 studies had high risk of bias (16, 17, 23, 45–47, 49, 51–53, 59, 60, 62, 66, 67, 69, 70, 73, 76–78, 80).

### Effect of L-carnitine supplementation on FBG (mg/dl) and subgroup analysis

**Figure 2:**
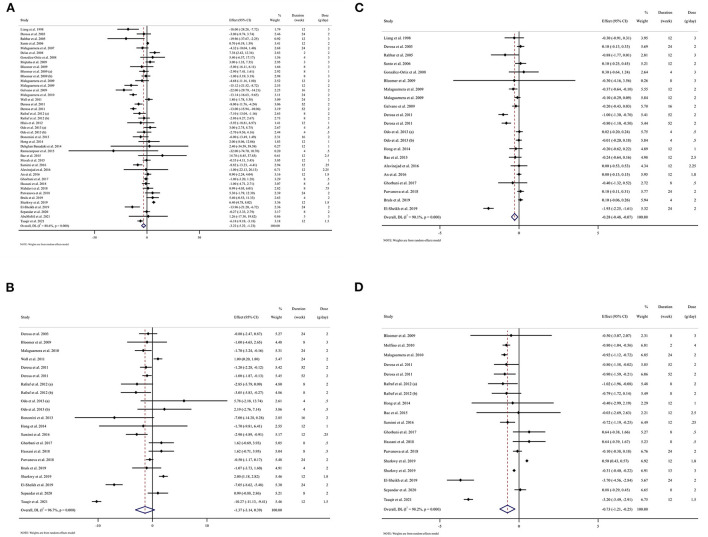
Forest plot detailing weighted mean difference and 95% confidence intervals (CIs) for the effect of L-carnitine consumption on **(A)** FBG (mg/dl); **(B)** Insulin (pmol/l); **(C)** HbA1c (%); **(D)** HOMA-IR. FBG, fasting blood glucose; HOMA-IR, homeostasis model assessment for insulin resistance; hemoglobin A1C, HbA1C; CI, confidence interval, weighted mean difference; WMD. Horizontal lines represent 95% of CIs. Diamonds represent pooled estimates from the random-effects analysis.

L-carnitine supplementation had a significant effect on FBG in the intervention compared to the placebo group (Figure 2A). Subgroup analyses showed that L-carnitine supplementation had a lowering effect on FBG (mg/dl) in baseline FBG ≥100 mg/dl [WMD = −5.91 mg/dl; 95% CI, −8.84 to −2.99; *p* < 0.001; *I*^2^ = 88.6%, *p* < 0.001], trial duration ≥12 week [WMD = −5.81 mg/dl; 95% CI, −8.73 to −2.88; *p* < 0.001; *I*^2^ = 92.5%, *p* < 0.001], intervention dose ≥2 g/day [WMD = −4.73 mg/dl; 95% CI, −7.45 to −2.01; *p* = 0.001; *I*^2^ = 89.5%, *p* < 0.001], participants with overweight (baselin BMI 25–29.9 kg/m^2^) [WMD = −4.35 mg/dl; 95% CI, −7.76 to −0.94; *p* = 0.018; *I*^2^ = 90.2%, *p* < 0.001], participants with obesity (baseline BMI >30 kg/m^2^) [WMD = −4.83 mg/dl; 95% CI, −8.44 to −1.21; *p* = 0.021; *I*^2^ = 91.9%, *p* < 0.001], and diabetic patients [WMD = −6.55 mg/dl; 95% CI, −9.92 to −3.18; *p* < 0.001; *I*^2^ = 90.5%, *p* < 0.001].

Subgroup analyses indicated significant between-study heterogeneity in studies conducted in all subgroups that were probable sources of heterogeneity, except in normal BMI participants (baseline BMI 18.5–24.9 kg/m^2^) (*I*^2^ = 46.4%, *p* = 0.071) (Table 3).

**Table 3:** Subgroup analyses of L-carnitine on glycemic indices in adults.

	**Number of effect sizes**	**WMD (95%CI)**	***P*-value**	**Heterogeneity**		
				***P* heterogeneity**	***I* ^2^**	***P* between sub-groups**
**Subgroup analyses of carnitine on serum FBG (mg/dl)**						
Overall effect	42	−3.22 (−5.21, −1.23)	0.002	< 0.001	88.6%	
Baseline FBG (mg/dl)						
< 100	16	−0.40 (−2.94, 2.13)	0.755	< 0.001	85.6%	0.005
≥100	26	−5.91 (−8.84, −2.99)	< 0.001	< 0.001	88.6%	
Trial duration (week)						
< 12	17	0.01 (−1.68, 1.69)	0.997	0.028	43.7%	0.001
≥12	25	−5.81 (−8.73, −2.88)	< 0.001	< 0.001	92.5%	
Intervention dose (g/day)						
< 2	15	−1.13 (−4.05, 1.79)	0.448	< 0.001	85.5%	0.077
≥2	27	−4.73 (−7.45, −2.01)	0.001	< 0.001	89.5%	
Baseline BMI (kg/m^2^)						
Normal (18.5–24.9)	8	0.92 (−1.96, 3.82)	0.529	0.071	46.4%	0.029
Overweight (25–29.9)	22	−4.35 (−7.76, −0.94)	0.018	< 0.001	90.2%	
Obese (>30)	11	−4.83 (−8.44, −1.21)	0.021	< 0.001	91.9%	
Health status						
Diaberic	17	−6.55 (−9.92, −3.18)	< 0.001	< 0.001	90.5%	0.017
Non-diabetic	25	−1.35 (−3.95, 1.24)	0.307	< 0.001	86.3%	
**Subgroup analyses of carnitine on serum Insulin (pmol/l)**						
Overall effect	21	−1.37 (−3.14, 0.39)	0.128	< 0.001	96.7%	
Trial duration (week)						
< 12	9	−0.01 (−1.43, 1.41)	0.988	0.033	52.1%	0.083
≥12	12	−2.33 (−4.53, −0.13)	0.038	< 0.001	97.9%	
Intervention dose (g/day)						
< 2	8	−0.39 (−5.51, 4.71)	0.879	< 0.001	98.5%	0.678
≥2	13	−1.50 (−2.53, −0.47)	0.004	< 0.001	86.3%	
Baseline BMI (kg/m^2^)						
Overweight (25–29.9)	13	−0.05 (−1.33, 1.22)	0.932	< 0.001	73.2%	0.011
Obese (>30)	7	−3.39 (−6.32, −0.46)	0.023	< 0.001	98.2%	
Health status						
Diaberic	9	−1.15 (2.35, 0.05)	0.061	< 0.001	87.0%	0.813
Non-diabetic	12	−1.57 (−4.84, 1.70)	0.346	< 0.001	97.9	
**Subgroup analyses of carnitine on serum HbA1c (%)**						
Overall effect	21	−0.27 (−0.47, −0.07)	0.007	< 0.001	90.1%	
Trial duration (week)						
< 12	6	0.04 (−0.05, 0.14)	0.400	0.839	0.0%	0.001
≥12	15	−0.40 (−0.65, −0.14)	0.002	< 0.001	92.1%	
Intervention dose (g/day)						
< 2	5	−0.01 (−0.11, 0.08)	0.772	0.813	0.0%	0.011
≥2	16	−0.38 (−0.64, −0.11)	0.005	< 0.001	92.0%	
Baseline BMI (kg/m^2^)						
Overweight (25–29.9)	15	−0.08 (−0.17, 0.01)	0.091	0.168	25.4%	0.064
Obese (>30)	5	−0.75 (−1.37, −0.13)	0.018	< 0.001	96.4%	
Health status						
Diaberic	15	−0.41 (−0.69, −0.13)	0.004	< 0.001	92.5%	0.010
Non-diabetic	6	−0.02 (−0.11, 0.07)	0.634	0.833	0.0%	
**Subgroup analyses of carnitine on HOMA-IR**						
Overall effect	18	−0.73 (−1.21, −0.25)	0.003	< 0.001	98.2%	
Trial duration (week)						
< 12	7	−0.26 (−0.80, 0.27)	0.329	< 0.001	77.3%	0.075
≥12	11	−1.00 (−1.60, −0.39)	0.001	< 0.001	98.8%	
Intervention dose (g/day)						
< 2	6	−0.44 (−2.21, 1.32)	0.623	< 0.001	99.2%	0.709
≥2	12	−0.78 (−1.11, −0.45)	< 0.001	< 0.001	89.5%	
Baseline BMI (kg/m^2^)						
Overweight (25–29.9)	10	−0.15 (−0.77, 0.46)	0.618	< 0.001	96.8%	0.044
Obese (>30)	18	−1.28 (−2.19, −0.37)	0.006	< 0.001	98.1%	
Health status						
Diabetic	8	−0.73 (−1.34, −0.11)	0.020	< 0.001	89.7%	0.983
Non diabetic	10	−0.74 (−1.39, −0.08)	0.027	< 0.001	98.9%	

BMI, body mass index; CI, confidence interval; FBG, fasting blood glucose; HbA1c, hemoglobin A1c; HOMA-IR, homeostatic model assessment for insulin resistance; WMD, weighted mean differences.

### Effect of L-carnitine supplementation on serum insulin (pmol/l) and subgroup analysis

L-carnitine supplementation had not a significant effect on insulin (Figure 2B). Subgroup analyses showed that L-carnitine supplementation had a reduction effect on insulin (pmol/l) in trial duration ≥12 week [WMD = −2.33 pmol/l; 95% CI, −4.53 to −0.13; *p* = 0.038; *I*^2^ = 97.9%, *p* < 0.001], intervention dose ≥2 g/day [WMD = −1.50 pmol/l; 95%CI, −2.53 to −0.47; *p* = 0.004; *I*^2^ = 86.3%, *p* < 0.001], and participants with obesity (baseline BMI >30 kg/m^2^) [WMD = −3.39 pmol/l; 95%CI, −6.32 to −0.46; *p* = 0.023; *I*^2^ = 98.2%, *p* < 0.001].

Subgroup analyses indicated significant between-study heterogeneity in studies conducted in all subgroups that were not probable sources of heterogeneity (Table 3).

### Effect of L-carnitine supplementation on serum HbA1c (%) and subgroup analysis

L-carnitine supplementation had a significant effect on HbA1c (Figure 2C). Subgroup analyses showed that L-carnitine supplementation had a lowering effect on HbA1c (%) in trial duration ≥12 week [WMD = −0.40 %; 95% CI, −0.65 to −0.14; *p* = 0.002; *I*^2^ = 92.1%, *p* < 0.001], intervention dose ≥2 g/day [WMD = −0.38 %; 95% CI, −0.64 to −0.11; *p* = 0.005; *I*^2^ = 92.0%, *p* < 0.001], participants with obesity (baselin BMI >30 kg/m^2^) [WMD = −0.75 %; 95% CI, −1.37 to −0.13; *p* = 0.018; *I*^2^ = 96.4%, *p* < 0.001] and diabetic patients [WMD = −0.41 %; 95% CI, −0.69 to −0.13; *p* = 0.004; *I*^2^ = 92.5%, *p* < 0.001].

Subgroup analyses indicated no significant between-study heterogeneity in studies conducted in the trial duration <12 week (*I*^2^ = 0.0%, *p* = 0.839), intervention dose <2 g/day (*I*^2^ = 0.0%, *p* = 0.813), participants with overweight (*I*^2^ = 25.4%, *p* = 0.168) and non-diabetic participants (*I*^2^ = 0.0%, *p* = 0.833) that were probable sources of heterogeneity (Table 3).

### Effect of L-carnitine supplementation on HOMA-IR and subgroup analysis

Carnitine supplementation had a significant effect on HOMA-IR (Figure 2D). We conducted the subgroup analyses which showed that L-carnitine supplementation had a lowering effect on HOMA-IR in trial duration ≥12 week [WMD = −1.00; 95% CI, −1.60 to −0.39; *p* = 0.001; *I*^2^ = 98.8%, *p* < 0.001], intervention dose ≥2 g/day [WMD = −0.78; 95% CI, −1.11 to −0.45; *p* < 0.001; *I*^2^ = 89.5%, *p* < 0.001], participants with obesity (baseline BMI >30 kg/m^2^) [WMD = −1.28; 95% CI, −2.19 to −0.37; *p* = 0.006; *I*^2^ = 98.1%, *p* < 0.001], diabetic patients [WMD = −0.73; 95% CI, −1.34 to −0.11; *p* = 0.020; *I*^2^ = 89.7%, *p* < 0.001] and non-diabetic patients [WMD = −0.74; 95% CI, −1.39 to −0.08; *p* = 0.028; *I*^2^ = 89.9%, *p* < 0.001].

Subgroup analyses showed significant between-study heterogeneity in studies conducted in all subgroups that were not probable sources of heterogeneity (Table 3).

### Publication bias

**Figure 3:**
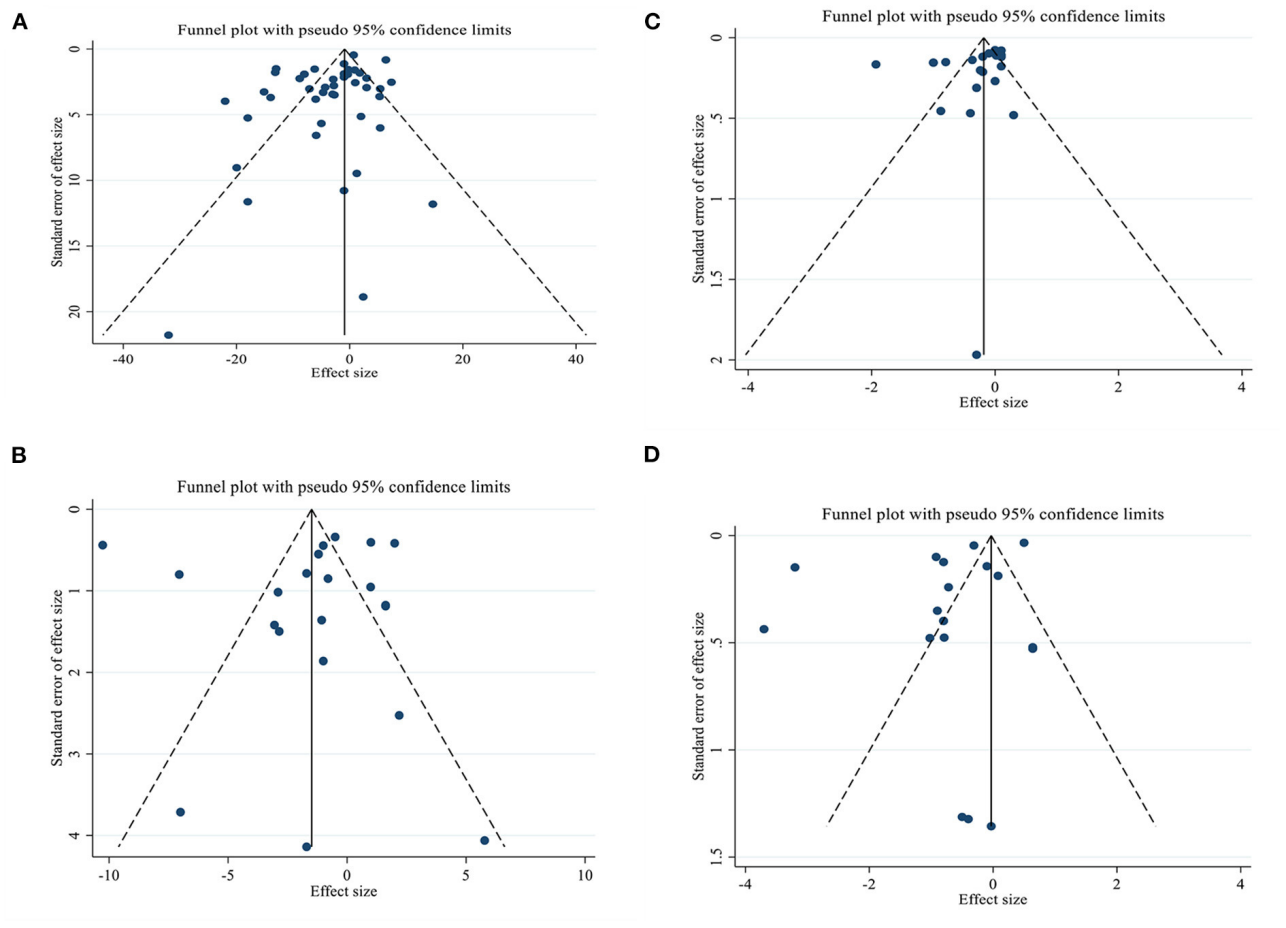
Funnel plots for the effect of L-carnitine consumption on **(A)** FBG (mg/dl); **(B)** Insulin (pmol/l); **(C)** HbA1c (%); **(D)** HOMA-IR. FBG, fasting blood glucose; HOMA-IR, homeostasis model assessment for insulin resistance; HbA1C, hemoglobin A1C.

Although the visual inspection of funnel plots showed slight asymmetries, no significant publication bias was detected for insulin according to Begg ($P_{\text{Beg}{\text{g}}^{\text{′}}\text{stest}}$ = 0.204) and Egger's test ($P_{\text{Egge}{\text{r}}^{\text{′}}\text{stest}}$ = 0.995; Figure 3B). The statistical test showed evidence of a publication bias for HbA1c ($P_{\text{Beg}{\text{g}}^{\text{′}}\text{stest}}$ = 0.027, $P_{\text{Egge}{\text{r}}^{\text{′}}\text{stest}}$ = 0.129; Figure 3C), and HOMA-IR ($P_{\text{Beg}{\text{g}}^{\text{′}}\text{stest}}$ = 0.046, $P_{\text{Egge}{\text{r}}^{\text{′}}\text{stest}}$ = 0.060; Figure 3D), for FBG ($P_{\text{Beg}{\text{g}}^{\text{′}}\text{stest}}$ = 0.599, $P_{\text{Egge}{\text{r}}^{\text{′}}\text{stest}}$ = 0.014; Figure 3A).

### Non-linear dose-response analysis

**Figure 4:**
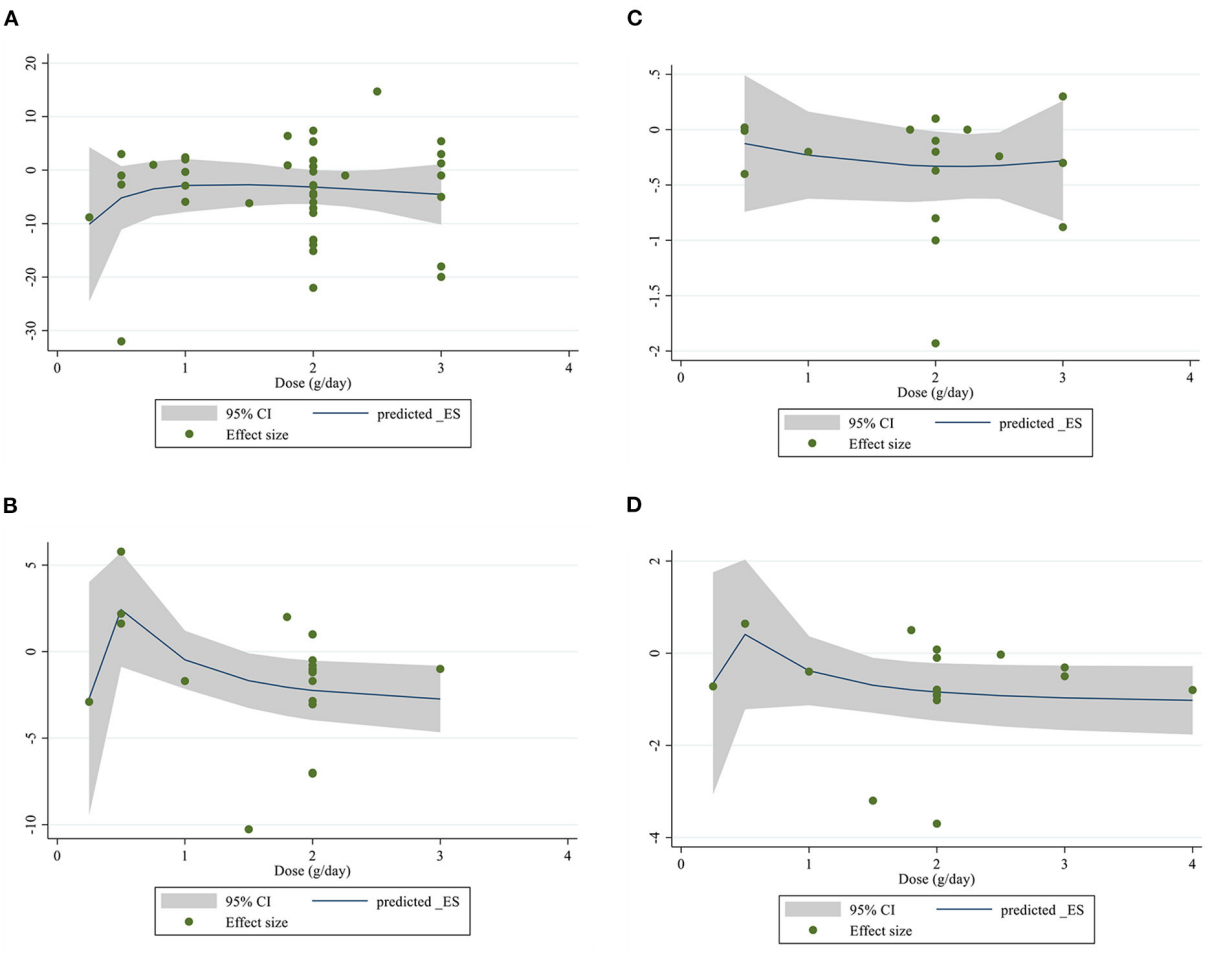
Non-linear dose-response relations between L-carnitine consumption and absolute mean differences. Dose-response relations between dose (mg/day) and absolute mean differences in **(A)** FBG (mg/dl); **(B)** Insulin (pmol/l); **(C)** HbA1c (%); **(D)** HOMA-IR. FBG, fasting blood glucose; HOMA-IR, homeostasis model assessment for insulin resistance; HbA1C, hemoglobin A1C.

**Figure 5:**
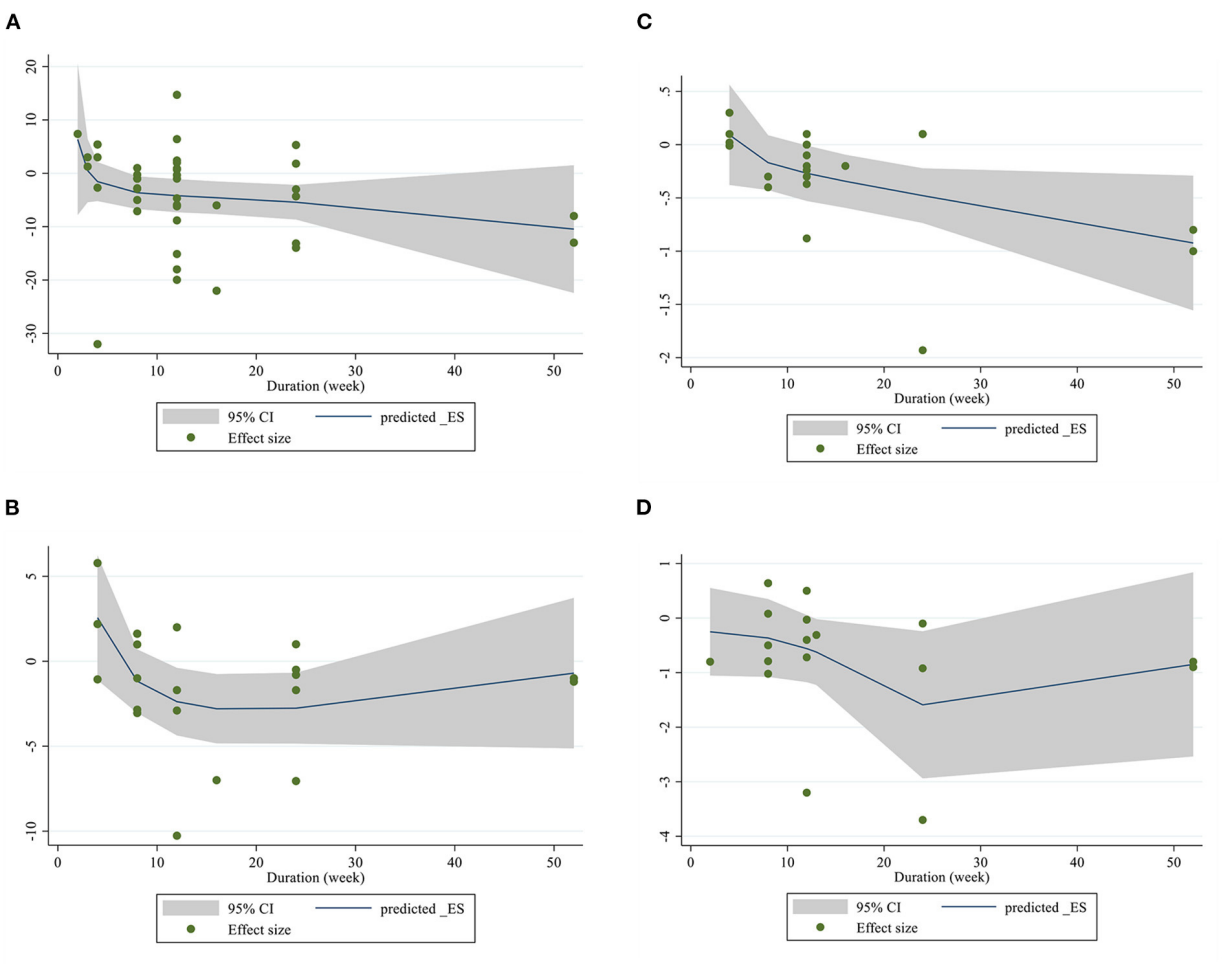
Non-linear dose-response relations between L-carnitine consumption and absolute mean differences. Dose-response relations between duration of intervention (week) and absolute mean differences in **(A)** FBG (mg/dl); **(B)** Insulin (pmol/l); **(C)** HbA1c (%); **(D)** HOMA-IR. FBG, fasting blood glucose; HOMA-IR, homeostasis model assessment for insulin resistance; HbA1C, hemoglobin A1C.

For the dose-response analysis between L-carnitine supplementation and FBG, insulin, HOMA-IR, and HbA1c, we used a one-stage non-linear dose-response analysis. We did not find a significant non-linear relationship between dose (g/day) (coefficients = −1.72, *p* = 0.154) and changes in FBG, but we found a significant non-linear relationship (coefficients = 7.45, *p* = 0.020) between duration of intervention and changes in FBG, It seems that the effective duration for decreasing of FBG is 50 weeks (Figures 4A, 5A). In addition, there was a significant non-linear relationship between dose (coefficients = 2.82, *p* = 0.020) and changes in insulin. Dose ≥2 g/day is more effective for decreasing insulin level, but we did not find a significant non-linear relationship between the duration of the intervention (weeks) (coefficients = 0.74, *p* = 0.177) and changes in insulin (Figures 4B, 5B). Also, we did not find a significant non-linear relationship between dose (g/day) (coefficients = −7.25, *p* = 0.140) and changes in HbA1c, although there was a significant non-linear relationship between duration of intervention (coefficients = −0.07, *p* < 0.001) and changes in HbA1c. It seems that the optimal duration for HbA1c reduction is 50 weeks (Figures 4C, 5C). In addition, we did not find a significant non-linear relationship between dose (g/day) (coefficients = −8.54, *p* = 0.054) and changes in HOMA-IR, although there was a significant non-linear relationship between duration of the intervention (coefficients = −0.22, *p* = 0.015) and changes in HOMA-IR; the prominent duration for decreasing of HOMA-IR seems to be 50 weeks (Figures 4D, 5D).

### Meta-regression analysis

**Figure 6:**
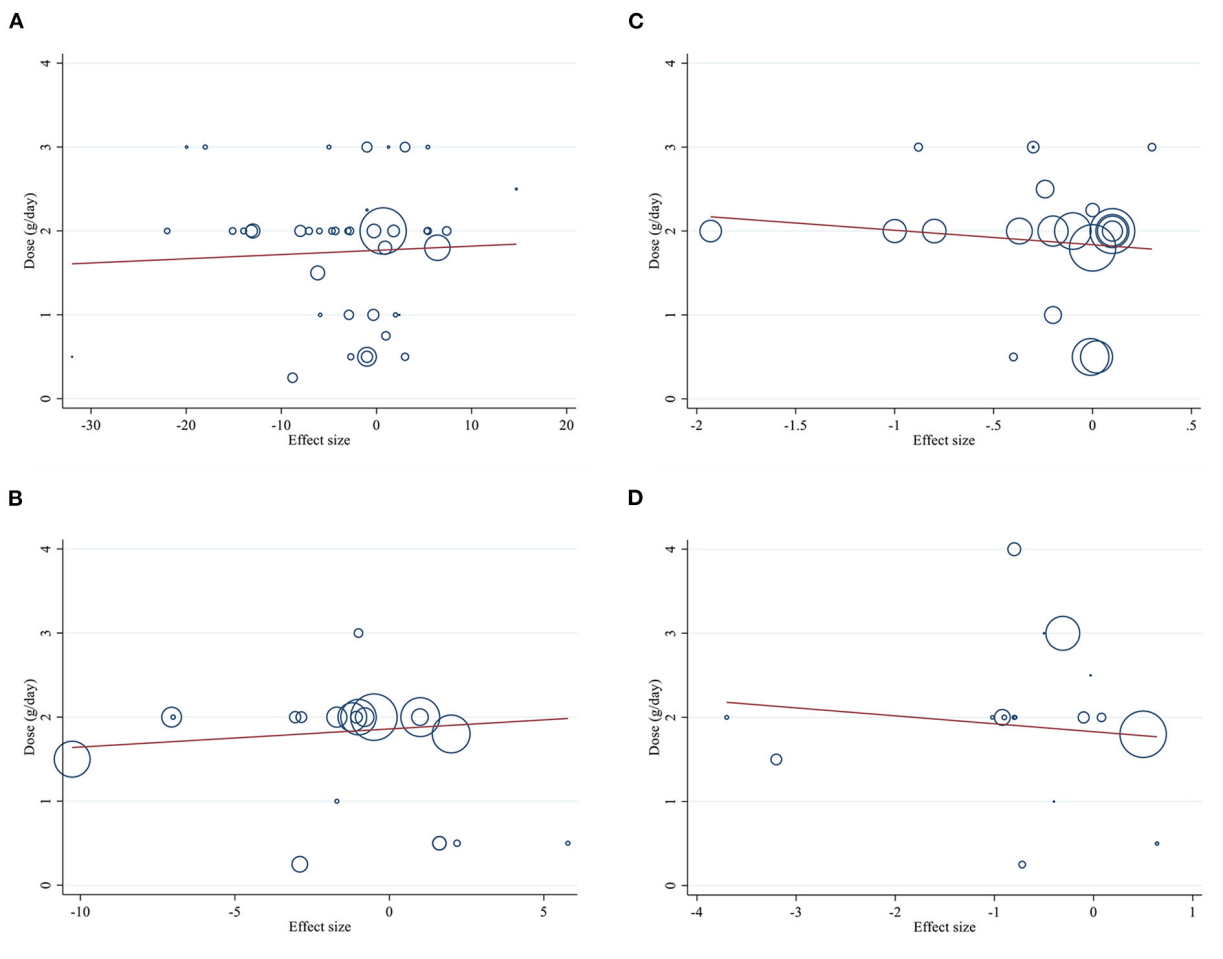
Linear relations between L-carnitine consumption and absolute mean differences. Dose-response relations between dose (mg/day) and absolute mean differences in **(A)** FBG (mg/dl); **(B)** Insulin (pmol/l); **(C)** HbA1c (%); **(D)** HOMA-IR. FBG, fasting blood glucose; HOMA-IR, homeostasis model assessment for insulin resistance; HbA1C, hemoglobin A1C.

**Figure 7:**
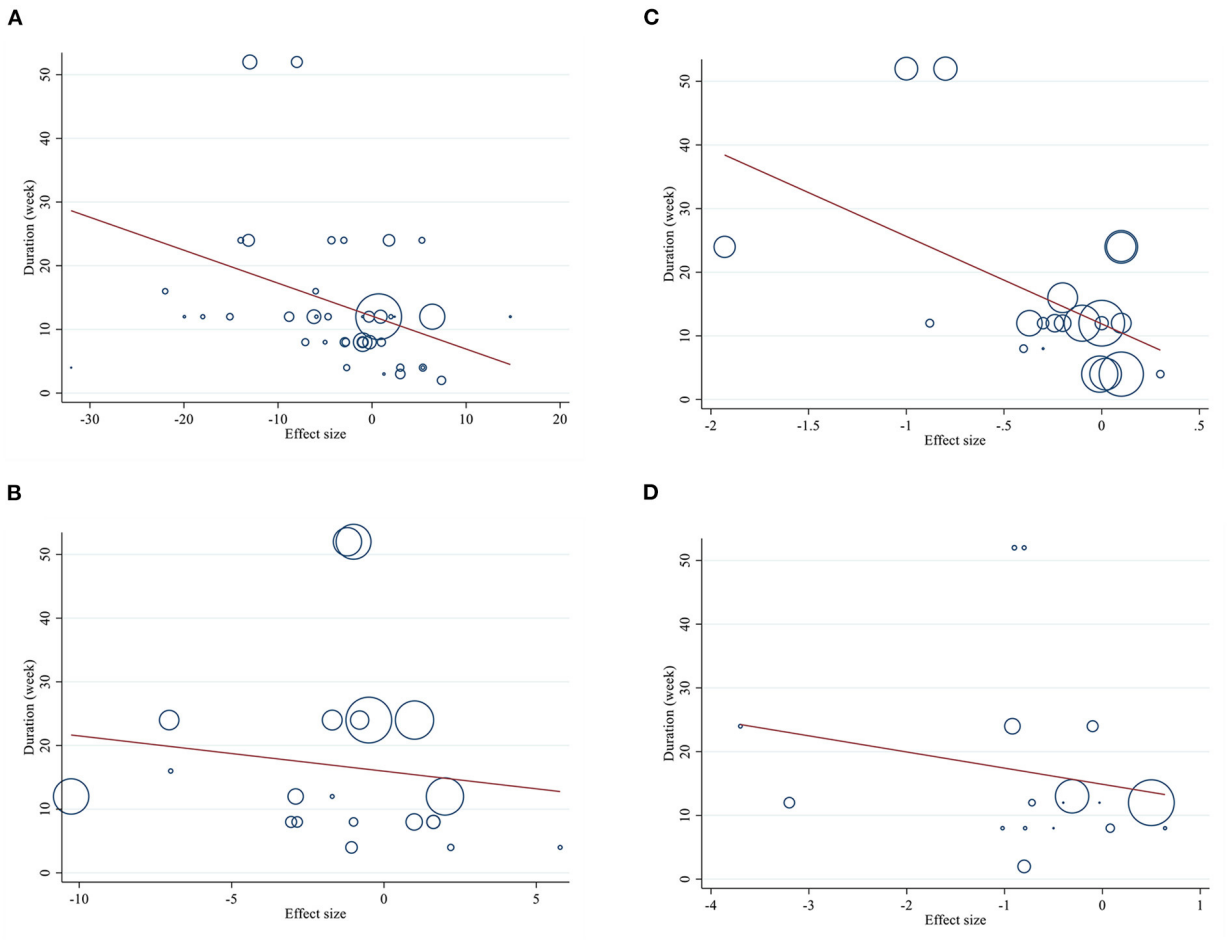
Linear relations between L-carnitine consumption and absolute mean differences. Dose-response relations between duration of intervention (week) and absolute mean differences in **(A)** FBG (mg/dl); **(B)** Insulin (pmol/l); **(C)** HbA1c (%); **(D)** HOMA-IR. FBG, fasting blood glucose; HOMA-IR, homeostasis model assessment for insulin resistance; HbA1C, hemoglobin A1C.

Meta-regression analyses were performed to assess whether FBG, insulin, HOMA-IR, and HbA1C was affected by L-carnitine doses and intervention durations. We did not find a significant linear relationship between dose (g/day) (coefficients = 0.01, *p* = 0.951) and changes in FBG, but there was a significant linear relationship between duration (weeks) (coefficients = −0.53, *p* = 0.031) of intervention and changes in FBG (Figures 6A, 7A). In addition, there was no significant linear relationship between dose (g/day) (coefficients = 0.02, *p* = 0.600) and duration of intervention (weeks) (coefficients = −0.54, *p* = 0.578) and changes in insulin (Figures 6B, 7B). Also, we did not find a significant linear relationship between dose (g/day) (coefficients = −0.16, *p* = 0.586) and changes in HbA1C, although there was a significant linear relationship between duration (weeks) (coefficients =-16.45, *p* = 0.016) of intervention and changes in HbA1C (Figures 6C, 7C). We did not find a significant linear relationship between dose (g/day) (coefficients = −0.09, *p* = 0.652) and duration (weeks) of intervention (coefficients = −2.68, *p* = 0.433) and changes in HOMA-IR (Figures 6D, 7D).

### GRADE assessment

The GRADE evidence profile and the certainty in outcomes of L-carnitine supplementation on FBG, insulin, HOMA-IR, and HbA1c were shown in Table 4. The quality of evidence was low due to severe inconsistencies and risk of bias for FBG, inconsistency, imprecision for insulin, and inconsistency and publication bias for HOMA-IR. Although the quality of evidence was moderate due to inconsistency, the risk of bias and publication bias for HbA1c.

**Table 4:** GRADE profile of L-carnitine for glycemic indices.

**Outcomes**	**Risk of bias**	**Inconsistency**	**Indirectness**	**Imprecision**	**Publication bias**	**WMD (95%CI)**	**Quality of evidence**
FBG	Serious limitation	Very serious limitation^a^	No serious limitation	No serious limitation	No serious limitation	−3.22 (−5.21, −1.23)	⊕⊕ Low
Insulin	No serious limitation	Very serious limitation^a^	No serious limitation	Serious limitation^b^	No serious limitation	−1.37 (−3.14, 0.39)	⊕⊕ Low
HbA1c	Serious limitation	Very serious limitation^a^	No serious limitation	No serious limitation	Serious limitation	−0.27 (−0.47, −0.07)	⊕⊕⊕ Moderate
HOMA-IR	No serious limitation	Very serious limitation^a^	No serious limitation	No serious limitation	Serious limitation	−0.73 (−1.21, −0.25)	⊕⊕ Low

a There is significant heterogeneity for FBG (I^2^ = 88.5%), Insulin (I^2^ = 96.5%), HbA1C (I^2^ = 90.5%) and HOMA-IR (I^2^ = 98.1).

b There is no evidence of significant effects of carnitine consumption on Insulin.

### Sensitivity analysis

According to the sensitivity analysis, no study affected the overall results after removing individual study effects.

## Discussion

Present dose-response meta-analysis revealed that L-carnitine intake has beneficial effects on glycemic indices by reducing FBG, HbA1c, and HOMA-IR, whereas insulin levels were not changed in the overall analysis. Also; we found significant associations between L-carnitine supplementation and glycemic indices change in the highest vs. lowest duration in the non-linear dose-response analysis.

Our findings identified that the optimal duration for reducing FBG, HbA1c and HOMA-IR is 50 weeks, although this change was not significant for insulin levels. Insulin changes were significant with the increase of L-carnitine in highest vs. lowest doses and results showed that doses higher than 2 mg/day are more effective on reducing insulin levels.

Currently, the decrease in the level of physical activity, sedentary lifestyle, and unhealthy diets have caused an increase in obesity and overweight, as well as glycemic indices disorders and ultimately its progression to type 2 diabetes (T2D) (81, 82). It seems that nutritional supplements can be effective in preventing or treating chronic diseases, especially in subjects who are at risk of deficiency (83, 84).

In Asadi et al., meta-analysis study; L-carnitine intake was related to a significant reduction in fasting plasma glucose, HbA1c, and HOMA-IR in individuals with cardiovascular risk factors in comparison with control groups (25), the results of this study confirm the findings of our meta-analysis. Although Asadi et al.'s study had some differences from the present study including the type of participants that our study was conducted on all healthy and unhealthy individuals, while this study was conducted only on subjects at risk of cardiovascular diseases, the number of included studies was higher in our study (41 studies vs. 24 articles) and also unlike of this study dose-response analysis was done in our meta-analysis. Also, in another meta-analysis, it was shown that L-carnitine can reduce glycemic indices such as FBG, HbA1c, HOMA-IR, and insulin (24), although this study differs from our study in terms of significant changes in insulin levels, this observed controversy may be justified by the different quality and also the number of included studies. An interventional study conducted by Hadadinezhad et al. showed that L-carnitine supplementation with a dose of 3 g/day for 12 weeks in patients with T2D significantly reduced FBG, but had no significant effect on HbA1c and 2-h post-prandial blood glucose (85). It should be kept in mind that one of the reasons for the difference between the results of this study and our research in terms of HbA1c is that our study was conducted on all people with and without diabetes, while this study was conducted on participants with at least 8 years of history of diabetes and, therefore; since HbA1c levels represent blood glucose levels in the last 2–3 months, probably 12 weeks of intervention cannot have a significant effect on HbA1c levels in subjects with a long history of diabetes. Also; the use of different doses of carnitine is another reason for these contradictions (86). A meta-analysis study showed that for L-carnitine to have significant effects on FBG levels in patients with diabetes, 2 grams/day should be taken as a supplement for at least 36 weeks, also it is estimated that 2 g of L-carnitine per day is needed for at least 106 weeks to have a significant effect on HbA1c levels (87). One of the main differences between our study and Wang et al. (87) research was the number of included studies, which was 41 in our study vs. 8 articles, and these studies were only conducted on subjects with diabetes, while the studies included in our research were done on all individuals. Furthermore, in another clinical trial study, Liang et al. (23) showed that L-carnitine supplementation for 12 weeks at dose of 3 grams/day had no significant effect on FBG and HbA1c in diabetic patients. The results of this study do not confirm our findings, because carnitine seems to be taken up by muscles and liver, and this process is regulated by insulin and glucagon hormones, while; this study was conducted in subjects with diabetes, whose levels of these hormones are disturbed (88).

The main function of L-carnitine is probably to increase the fatty acids oxidation by transporting long-chain fatty acids from the cytosol to the mitochondrial matrix. But there are controversial reports regarding the effect of fatty acids oxidation on glucose metabolism. One hypothesis proposes that muscle insulin resistance results from decreased mitochondrial fatty acids oxidation. In such a way that unoxidized fatty acids are rerouted toward the synthesis of diacylglycerol and ceramide, which in turn stimulate stress-induced protein kinases that inhibit insulin signaling (89, 90). While, Randle et al. suggested that increased fatty acid oxidation inhibits glucose utilization in muscle. Inhibition of glucose utilization by fatty acids is a type of glucose intolerance that may lead to insulin resistance. In fact, fatty acid oxidation metabolites inhibit several glycolytic steps such as glucose transport and phosphorylation, 6-phosphofructo-1-kinase (PFK-1), and pyruvate dehydrogenase (91).

Despite these two hypotheses; some study reported that L-carnitine supplementation may affect insulin receptors and increase their sensitivity (92, 93). It is also reported that L-carnitine supplementation can improve the glycemic status and related indices by changing the expression of genes related to glycolytic and gluconeogenic enzymes, modulating the activity of the pyruvate dehydrogenase enzyme complex, and changing the expression of genes involved in insulin metabolism (94, 95).

Also, oxidative stress causes a disturbance in the function of pancreatic beta cells, and L-carnitine can improve the function of these cells and increase their efficiency by reducing the level of oxidative stress (67, 96). However, it seems that long-term L-carnitine intake eventually converts to a metabolite called trimethylamine N-oxide (TMAO) (97–99), and increased circulating levels of this metabolite can increase the prevalence of diabetes (54% per 5 μmol/L increment of plasma TMAO) (100), nonetheless one study showed that increased TMAO plasma level by 30 μmol/L after 24 weeks of L-carnitine supplementation was not related to glucose, insulin and HOMA-IR levels changes (97).

In the subgroup analysis, our results revealed that baseline FBG, trial duration, intervention dose, BMI, and health status had a significant effect on L-carnitine effects on FBG levels. Also, trial duration, intervention dose, individuals with obesity (BMI ≥ 30), and diabetic patients were among the factors that caused significant effects of L-carnitine on HbA1c levels. Effects of L-carnitine on FBG were significant in doses equal to or higher than 2 g/day and 12 weeks or more duration in subjects with overweight and obesity. Based on the results of previous studies, it seems that L-carnitine needs a longer duration of use to have significant effects on glycemic indices-related markers in diabetic patients (87). In an interventional study, it was shown that L-carnitine supplementation with a dose of 3 g/day for 12 weeks significantly reduced FBG, but its effects on HbA1c reduction were not significant in diabetic patients (17), the results of this study confirm our findings in terms of reducing FBG, but from the point of view of the effect on HbA1c, it does not confirm the results of our study. In term of HbA1c, considering that in this study the average BMI of the participants is in the overweight range and our results also showed that the effects of L-carnitine on HbA1c is significant only in subjects with obesity, so it can be said that this study also agrees with our findings.

Subgroup analysis of our findings showed that trial duration, intervention dose, and BMI status significantly affect the insulin level, as well as, it was shown that trial duration, intervention dose, obesity, and health status have a significant effect on the HOMA-IR levels. In line with our findings; A systematic review study conducted by Maleki et al. showed that L-carnitine supplementation in women with polycystic ovary syndrome (PCOs) improves glycemic status and reduces insulin resistance (101) which can be due to the increase in basal metabolic rate and lean body mass, as well as weight loss and improvement of related indices (102, 103). In one meta-analysis study in 2017, Xu et al. confirmed the findings of our study and showed that L-carnitine supplementation can have significant effects on HOMA-IR (104). It seems that serum carnitine levels decrease in individuals with obesity and metabolic syndrome due to insulin resistance, therefore supplement therapy with L-carnitine can help improve glycemic status by reducing insulin resistance (104). The results of Wutzke et al.'s study showed that taking an L-carnitine supplement at a dose of 3 g/day for 10 days, although it increased fat oxidation, did not have a significant effect on body weight and other factors, which in a way confirms the results of our study. Because doses of L-carnitine have mostly had significant effects for more than 12 weeks (105). It seems that L-carnitine can have optimal and significant effects on human health when it is accompanied by increased physical activity, modification of lifestyle, and compliance with a healthy diet (106–109). Therefore, it can be said that L-carnitine should be consumed at a dose of more than 2 g/day for more than 12 weeks and often in individuals with obesity to have significant effects on insulin and HOMA-IR levels.

The non-linear dose-response analysis revealed a significant negative relationship between FBG levels and L-carnitine intervention duration for 4 weeks and more. Moreover, HbA1c and HOMA-IR levels decreased significantly after about ≥12 and ≥8 weeks of L-carnitine supplementation, respectively. But it was not significant for insulin levels. It can be said that the optimal duration for effective reduction of FBG, HOMA-IR, and HbA1c was 50 weeks. Although unlike the other three indices insulin changes were significant with the increase of L-carnitine in highest vs. lowest doses and results showed that an optimal dose of about 2 g/day is more effective for decreasing insulin levels.

Of course, our study has some limitations, including that most of the included articles showed high bias and heterogeneity which makes it difficult to reach a definitive conclusion about the effects of carnitine. Although we tried to find the source of the heterogeneity by performing subgroup analysis. Moreover, we did not evaluate the effects of other glycemic indices such as 2-h post-prandial glucose due to the lack of examination of this outcome in clinical trials.

Although all studies used randomization; information on allocation concealment, randomization efficiency, and withdrawal was not consistently disclosed. Moreover, there are differences in laboratory assessment methods in different trials, as well as differences in intra assay coefficient of variation (intra-assay CV) and inter-assay coefficient of variation (inter-assay CV). Although adverse events were mentioned in some trials, most of them were not reported.

There are also strengths in the present study. To our knowledge, the present study is one of the first comprehensive dose-response meta-analyses to evaluate the L-carnitine effects on glycemic markers in diabetes and non-diabetic adults and we considered all published RCTs that were conducted on the effect of L-carnitine sapplementation on glycemic indices. Furthermore, we performed a dose-response analysis and considered different subgroups to evaluate the effects of L-carnitine on glycemic indices. All trials were included based on inclusion criteria, with varying individuals, which provides the possibility of subgroup analysis.

The randomized and placebo-controlled design of all included trials and the double-blind design of most of them can also be other strengths and due to the RCT nature of the studies, the drugs used by the patients (especially in diabetic patients), the diet and the level of physical activity of the participants were controlled and in fact their effects were considered and it can be said that the pure effects of L-carnitine was evaluated. In the current meta-analysis, there were no time and language restrictions for inclusion of studies. In addition, GRADE assessment, sensitivity tests, and subgroup analysis were used to assess quality of studies, detect publication bias and identify potential sources of heterogeneity among trials, respectively.

## Conclusions

The findings of our systematic review and dose-response meta-analysis showed a significant reductions for FBG, HbA1c, and HOMA-IR levels. However, based on our analysis, L-carnitine failed to significantly affect serum insulin. Moreover, dose-response analysis demonstrated that L-carnitine supplementation at doses of equal 2 g/day has an optimal effect on insulin levels. On the other hand; 50 weeks of intervention has beneficial effects on decreasing HOMA-IR, HbA1c, and FBG. Larger, well-designed trials are still required to further evaluation of this association.

## Data availability statement

The original contributions presented in the study are included in the article/supplementary material, further inquiries can be directed to the corresponding authors.

## Author contributions

MZ designed the study. MZ and OA developed the search strategy and assessed the risk of bias of the meta-analyses. MZ, MN-S, and OA extracted the data and conducted the analyses. NP, RG-E, NR, and SR drafted the manuscript. FS, OA, and MN-S interpreted the results. FS, OA, and SR revised manuscript. All authors read and approved the final manuscript.
